# Microservice Deployment Based on Multiple Controllers for User Response Time Reduction in Edge-Native Computing

**DOI:** 10.3390/s25103248

**Published:** 2025-05-21

**Authors:** Zhaoyang Wang, Jinqi Zhu, Jia Guo, Yang Liu

**Affiliations:** 1 School of Computer and Information Engineering, Tianjin Normal University, Tianjin 300387, China; 2310090021@stu.tjnu.edu.cn (Z.W.); c04s316@bupt.cn (J.G.); 002909@tjnu.edu.cn (Y.L.); 2 Yangtze Delta Region Institute (Quzhou), University of Electronic Science and Technology of China, Quzhou 324000, China

**Keywords:** microservice deployment, service mesh, multiple controllers, user response time

## Abstract

Microservice deployment methods in edge-native computing environments hold great potential for minimizing user application response time. However, most existing studies overlook the communication overhead between microservices and controllers, as well as the impact of microservice pull time on user response time. To address these issues, this paper proposes a multi-controller service mesh architecture to reduce data transfer overhead between microservices and controllers. Furthermore, we formulate the microservice deployment problem as an optimization problem aimed at minimizing both system communication overhead and microservice deployment cost. To achieve this, we introduce a novel RIME optimization algorithm and enhanced Adaptive Crested Porcupine Optimizer (RIME-ACPO) algorithm that optimizes microservice placement decisions. Notably, this algorithm incorporates a real-time resource monitoring-based load balancing algorithm, dynamically adjusting microservice deployment according to edge server resource utilization to enhance the execution performance of user applications. Finally, extensive simulation experiments were conducted to validate the effectiveness of the proposed algorithm. The experimental results demonstrate that, compared with other algorithms, our algorithm significantly improves user response time, optimizes resource utilization, and reduces the total cost.

## 1. Introduction

Cloud-native computing leverages modern technologies such as containerization, microservices, and automated management to create applications that are scalable, resilient, and efficient. In cloud-native computing, a single application is decoupled into many interdependent microservices, which are then merged and deployed as one or more containers. Decoupling applications into interdependent microservices contributes to improved scalability, flexibility, and overall performance [1]. This deployment approach not only enhances the flexibility of applications but also effectively handles the uncertainties of operations and evolution. The superior advantages of cloud-native computing have made it a primary cloud application deployment and the dominant paradigm in both centralized and edge cloud environments. Particularly, extending cloud-native computing to edge computing promotes the development of edge-native computing [2,3,4,5].

Recently, many researchers have studied in-depth microservices management and deployment in edge clouds. For example, leveraging the layered sharing benefits of microservices, the authors in [2] explored microservice placement and task offloading strategies for an edge cloud system, with the aim of enhancing the system’s overall throughput. The authors in [3] utilized an enhanced Lyapunov-based algorithm to improve system reliability while simultaneously meeting the delay demands of all user tasks. Reference [6] focused on achieving a balance between microservice download cost and microservice interaction cost within the layered architecture of microservices. To reduce the user delay and balance the network load, the authors in [7] used a reinforcement learning-based algorithm to optimize microservice deployment and routing in the edge cloud. To address the microservice placement challenge in edge computing, the authors in [8] employed an attention strategy to improve judgment processing.

**Figure 1:**
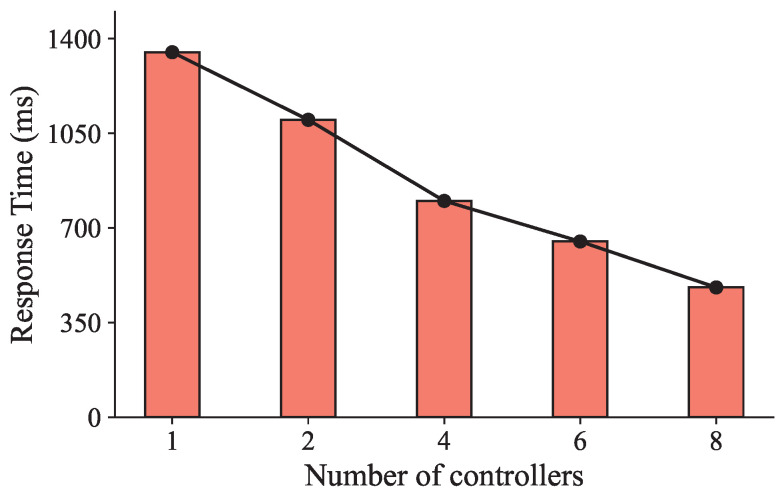
Comparison of user response time under different numbers of controllers.

However, after a careful review of the existing studies, we find that two main problems are ignored in most of the current studies. The first problem arises from the degraded user experience resulting from the increased communication delay between microservices and the controller in the single-controller service mesh architecture. Since the management of microservices in cloud-native computing is challenging [9], service mesh technology, which can effectively address communication-related issues among the controller and microservices, is proposed. Nevertheless, unlike the traditional central cloud, the edge cloud is characterized by a distributed and heterogeneous server architecture, consisting of a large number of widely dispersed edge servers with limited resources. Hence, deploying a single controller in the edge cloud as in the central cloud is unsuitable. This approach leads to increased communication latency between the controller and microservices, which, in turn, extends the user response time, particularly for delay-sensitive user requests. In addition, the single controller can become the system’s bottleneck, and its failure can cause system paralysis. To reduce the user response time, a potential solution is to deploy multiple controllers in the edge cloud. We conducted an experiment to verify the impact of the number of controllers on the user response time in an edge cloud with 20 edge servers. Each edge server was equipped with 4 cores, 8 GB of RAM, and a 500 GB disk. The user request rate was set to 500 requests per second. As shown in Figure 1, as the number of controllers rises, the user response time reduces. When the number of controllers reaches eight, the average user response time is only 0.53 times that of using a single controller, which indicates the significant impact of the number of controllers on user response time in edge-native computing.

The second issue pertains to the influence of microservices’ pull time on user response time. In cloud-native computing, microservices are generally placed in an on-demand fashion. This approach, which is a way of resource optimization, generates significant pull traffic and incurs high microservice deployment cost, resulting in additional startup delay and negatively impacting the user response time. This issue becomes more critical for user applications with real-time requirements. Therefore, reducing pull traffic and the associated microservice deployment cost is crucial for decreasing microservices’ pull time and minimizing user response time.

To tackle the aforementioned challenges, we propose a microservice deployment mechanism designed to enhance the performance of user application execution in edge-native computing environments. Firstly, multiple controllers are introduced. Compared to the traditional service mesh architecture with a single controller, the proposed architecture allows a microservice to communicate with the nearest controller. This markedly minimizes the data transmission latency between the microservice and the controller. Secondly, to guarantee the user application execution performance, we consider both the impact of the system communication overhead and the microservices’ pulling traffic based on the layered sharing characteristic of microservices. Finally, we formulate the deployment issue as a multi-objective optimization problem and propose a novel RIME optimization algorithm and enhanced Adaptive Crested Porcupine Optimizer (RIME-ACPO) algorithm to effectively solve the formulated problem. Experimental results show that the RIME-ACPO algorithm outperforms some other algorithms in terms of user response time, resource utilization, and total cost. The main contributions of this paper are as follows:

- To address the high communication overhead between microservices and controllers in distributed and heterogeneous edge-native computing environments, we propose a novel multi-controller-based service mesh architecture. This architecture is specifically designed to optimize system communication latency and enhance scalability by distributing control functions across multiple controllers.
- Building upon this architecture, we formulate microservice deployment as a multi-objective optimization problem aimed at simultaneously minimizing system communication overhead and microservice deployment cost to enhance user application execution performance.
- We propose a novel RIME-ACPO algorithm, which combines the RIME Optimization Algorithm (RIME) with an improved Adaptive Crested Porcupine Optimizer (ACPO) algorithm to obtain the optimal deployment decision. Notably, this algorithm incorporates a real-time resource monitoring-based load balancing algorithm, dynamically adjusting microservice deployment according to edge server resource utilization to enhance the execution performance of user applications.

The rest of the paper is organized as follows. The related works are presented in Section 2. The motivation is presented in Section 3. The system model is elaborated in Section 4. The calculation of communication overhead and deployment cost are analyzed in Section 5. Our proposed RIME-ACPO algorithm is described in Section 6. The dynamic load balancing algorithm is described in Section 7. The performance evaluation is demonstrated in Section 8. Finally, the conclusion is presented in Section 9.

## 2. Related Works

In cloud-native computing, container-based microservice deployment enables fast, flexible, and scalable service deployment. The deployment of container-based microservices has emerged as a prominent research focus. Microservices deployment strategies that meet diverse needs have been designed. In this section, we first introduce the research on service mesh and then describe the works related to microservice deployment.

### 2.1. The Service Mesh Technology

In cloud-native computing, service mesh technology provides an effective solution to the challenges associated with microservice communication, such as traffic routing, dependency management, load balancing, etc. A service mesh consists of a data plane and a control plane  [9]. Within the data plane, each microservice instance is accompanied by a sidecar. In the control plane, a centralized controller orchestrates network communication between microservices by interacting with these sidecars so as to simplify the complexity of microservice communication within the service mesh. Many researchers have already investigated the optimization of service mesh from various aspects. For example, to tackle the challenges of application management with the growing number of applications, the authors in [10] presented a security framework leveraging Istio and Kubernetes to construct a secure API service mesh. Further, an intelligent model was developed to associate new applications with pre-existing service mesh types. The authors in [11] addressed the security scheme in service mesh where controlling traffic might be exploited by application tenants. The security of the Istio-based service mesh was enhanced by separating the encrypted monitor and control traffic. To address the challenges of predicting microservice performance and estimating cloud application capacity, the authors in [12] introduced Capestor, a service mesh-based framework that simulates workloads, collects performance data, and uses ElasticNet regression to estimate microservice capacity with low prediction errors. Reference [13] proposed a case that applied provenance tracing within the service mesh to conduct the cross-layer prioritization of delay-sensitive requests. The authors in Reference [14] proposed CRG-based algorithms to optimize SDN controller placement for service-aware management in dynamic LEO satellite networks. The existing studies primarily concentrate on service mesh architectures that utilize a single controller. However, due to the heterogeneity and wide deployment scope of edge clouds, adopting a single controller, as used in central cloud environments, is impractical. This setup increases the communication latency between the controller and the microservice, leading to a rise in user response time. Li et al. [15] first proposed a service mesh architecture with several controllers deployed in the edge cloud. They only discussed how to place multiple controllers to minimize the communication cost between controllers and the services. Deploying microservices on servers as close as possible to the controllers can reduce the communication overhead between microservices and controllers. However, this study did not take into account the service time of user applications, which may lead to poor user application execution performance.

### 2.2. Microservice Deployment Optimization

For the optimization of microservice deployment, the authors in [16] used the BitTorrent for peer-to-peer (P2P) distribution of Docker images. This approach leveraged the bandwidth of all nodes in the cluster to reduce the load of the Docker Registry and substantially enhanced the distribution speed of Docker images. The authors in [17] suggested employing technology to enhance the efficiency in the process of Docker image construction. Meanwhile, through the use of a local file cache, without the intervention of interception from remote file requests, and the local provision of required files, the amount of downloaded traffic was largely decreased. Moreover, the authors in [18] introduced a distributed redundancy placement framework named SAARP, which reduced the download of redundant layers. Additionally, this study designed an iterative greedy algorithm to improve the deployment and processing efficiency of microservices in resource-constrained edge computing environments. The authors in [6] addressed the challenges posed by service dependencies during microservice deployment. A random integer-based microservice deployment strategy was designed to optimize the overall cost of microservice deployment in the edge cloud. A service mesh architecture with the distributed deployment of controllers was proposed in [15]; the aim of this paper was to deploy controllers with the purpose of minimizing overall control cost. The authors in Reference [19] used RMS_DDPG to optimize microservice deployment and routing, improving delay, load balancing, and robustness in mobile edge computing. The authors in Reference [20] presented a blockchain-based framework for secure, decentralized file sharing in edge computing and microservice environments. However, none of the aforementioned studies addressed key factors such as communication overhead between microservices, interaction overhead between microservices and controllers, and deployment costs of microservices and network load balancing when tackling the microservice placement problem. Neglecting these factors can lead to a decline in user application execution performance and negatively impact user experience. Hence, to enhance the performance of user applications and improve the overall user experience, we take these factors into account to optimize the placement of microservices.

### 2.3. The Multi-Controller Architecture of SDN

Considering the potential large-scale deployment of edge servers in the future, a single controller becomes inadequate for managing the service mesh when the number of edge servers significantly rises. Similar to the multi-controller architecture of SDN, employing multiple controllers in the edge cloud facilitates more effective management and control of widely distributed edge servers. Many existing studies have proposed the multi-controller architecture of SDN. For instance, in [21], the authors discussed how to achieve load balancing in a dynamic SDN environment by employing multiple controllers. The authors in [22] proposed a highly available multi-controller architecture and used an optimized k-means algorithm to place multiple controllers to reduce latency between the controller and its associated switch. The authors in [23] addressed scalability and reliability issues in SDN by proposing an optimized controller placement strategy in legacy networks, focusing on high-processing bandwidth nodes to improve controller load balancing and resiliency in large-scale SDN deployments. To improve both performance and resiliency, the authors in [24] introduced a multi-SDN controller architecture that leverages Open Network Operating System (ONOS) controllers to perform IP transformations. The authors in Reference [25] evaluated SDN controller architectures to optimize network performance, balancing throughput, memory usage, and jitter. Reference [26] proposed a deep learning-based switch migration strategy to optimize load balancing and reduce migration costs in SDN multi-controller environments.

The above studies provide valuable insights for addressing the problem of multiple controllers’ deployment in edge cloud environments. Taking into account both the heterogeneity and the widely distributed nature of edge servers in the edge computing environment, we thoroughly examine the impact of system communication overhead and microservices deployment cost to ensure optimal user application execution performance. Furthermore, a load balancing algorithm is proposed to further enhance the overall user experience.

## 3. Motivation

**Figure 2:**
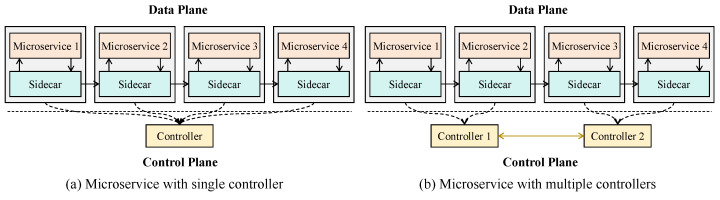
Microservices and controllers in edge-native computing.

Nowadays, many popular service mesh implementations, including Istio, Linked, and Consul, have been extensively studied. A traditional service mesh is typically composed of a data plane and a control plane [15], as shown in Figure 2a. In the data plane, each microservice instance has a sidecar next to it. In the data plane, a controller manages the communication among microservices by communicating with sidecars so that effective communication management of the cloud is achieved. However, the edge cloud is characterized by a distributed and heterogeneous server architecture consisting of a large number of widely dispersed edge servers with limited resources. Deploying multiple controllers can reduce the communication latency between controllers and microservices. As shown in Figure 2b, communication latency between controllers and microservices can be reduced by enabling each microservice to interact with its nearest controller. Further, the on-demand download approach of microservices leads to high deployment costs, which is particularly critical for user applications with real-time requirements. For example, in the matchmaking service of an online game, if certain microservices are not deployed, the cloud platform initiates the on-demand deployment, which may cause delays, especially during peak times with many uses. Hence, our goal is to optimize both the system communication overhead and the microservice deployment cost to minimize the user application execution time. As communication interactions between microservices significantly impact user response time [5,7], we consider the system communication overhead to consist of both the interaction delay among microservices and the data delivery delay between microservices and controllers.

**Figure 3:**
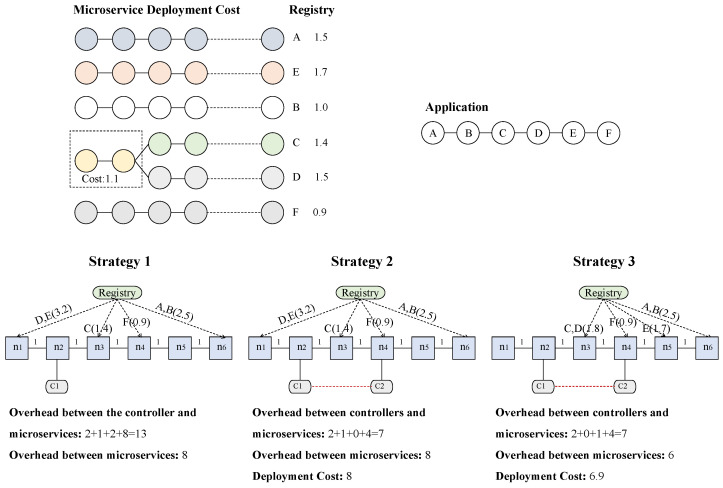
Deployment examples and comparisons.

To understand the necessity of deploying multiple controllers in the edge cloud and the issue of minimizing the microservices deployment cost and the communication overhead with the multi-controller architecture, we give an example as shown in Figure 3. In this instance, we consider an edge cloud with six edge servers and two controllers, namely $C_{1}$ and $C_{2}$. $C_{1}$ is deployed on server $n_{2}$ while $C_{2}$ is deployed on server $n_{4}$.

Suppose an application $APP_{1}$ with six microservices is deployed on the edge cloud. Let $APP_{1}$ = {*A*, *B*, *C*, *D*, *E*, *F*}, in which microservices *C* and *D* share the same layers with a sharing cost of 1.1 and *F* is placed on server $n_{4}$. Microservices of $APP_{1}$ need to communicate with each other. We assume *A* requires interaction with *B*, *B* requires communication with *C*, and *C* communicates with *D*, and so on. The costs of pulling different container images are shown in Figure 3. Given the limited capacity of server resources, we assume that each server can host a maximum of two microservices. Consider the following three different deployment strategies:

*Deployment Strategy 1*: This strategy only considers one controller. In this strategy, microservices *D* and *E* are deployed on $n_{1}$, *C* is deployed on $n_{3}$, and *A* and *B* are deployed on $n_{6}$. All microservices are managed by controller $C_{1}$. The interaction overheads between microservice *D* and controller $C_{1}$ and between *E* and $C_{1}$ are all 1. The interaction overhead between *C* and $C_{1}$ is 1, while the overhead between *F* and $C_{1}$ is 2. The interaction overhead between microservice *A* and $C_{1}$, as well as between microservice *B* and $C_{1}$, is 4. And the interaction between microservices is 8. Therefore, the total controller-to-microservice communication overhead is 13.

*Deployment Strategy 2*: In this strategy, we introduce another controller $C_{2}$ to the system. Microservices placed on servers $n_{1}$, $n_{2}$, and $n_{3}$ are managed by $C_{1}$ while microservices placed on servers $n_{4}$, $n_{5}$, and $n_{6}$ are managed by controller $C_{2}$. In this multi-controller system, the controller-to-microservice interaction overhead is reduced to 7 while the interaction cost between microservices remains 8. This demonstrates that introducing multiple controllers can effectively reduce the controller-to-microservice communication overhead.

*Deployment Strategy 3*: This strategy fully considers reducing the system communication overhead and the microservices deployment cost to enhance the user application execution performance under the multi-controller architecture. In this strategy, based on Deployment Strategy 2, *C* and *D* are deployed on server $n_{3}$ for layering sharing, and *E* is deployed on server $n_{5}$ to reduce the communication latency between microservice *E* and microservice *F*. Compared to the microservices deployment method of Strategy 2, the deployment cost and the system communication overhead, which consists of both the controller-to-microservice communication overhead and the communication overhead among microservices, are reduced to 6.9 and 13, respectively.

The above-mentioned instance shows us that a multi-controller architecture can significantly reduce the data transfer delay between the microservices and the controllers, especially when edge servers are widely distributed. Moreover, in the case of layer sharing, a carefully designed microservice deployment strategy can reduce the deployment cost and the system communication overhead, thereby improving the execution performance of user applications.

This paper proposes a microservice deployment mechanism specifically designed for latency-sensitive edge native computing applications. The solution primarily targets four representative scenarios: (1) intelligent traffic signal control systems requiring second-level vehicle-to-infrastructure response times, (2) campus security platforms demanding sub-second emergency alert propagation, (3) smart access control systems with second-level authentication latency, and (4) Industrial IoT (IIoT) predictive maintenance applications needing sub-second equipment monitoring. To address these requirements in distributed edge environments, our mechanism implements a tri-fold strategy: (1) hierarchical multi-controller coordination for enhanced control-plane efficiency, (2) joint optimization of system communication overhead and microservice deployment cost, and (3) dynamic load balancing—collectively fulfilling stringent user application requirements.

## 4. System Model

**Figure 4:**
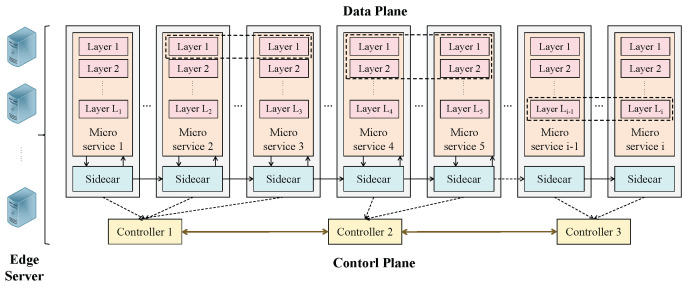
The proposed multi-controller system architecture.

The system’s structure is shown in Figure 4. The edge cloud is characterized as an undirected graph *G* = {*N*,*E*}, where *N* = {1, 2, …, *n*} is the set of edge servers and *E* denotes the set of edges between edge servers. The edge cloud adopts the service mesh technology. Suppose there is a number of *c* controllers in the system, and the set of controllers is *C* = {1, 2, 3, …, *c*}. A controller can be deployed on any edge server to effectively manage the communication between microservices. The controller centrally positioned among all controllers is selected to serve as the central controller for executing the following mentioned optimization and dynamic load balancing algorithms. In this multi-controller architecture, the sidecar interacts with the controller nearest to it to decrease the communication delay between microservices and controllers. For simplicity, we assume the location of the controllers is pre-deployed and fixed. Suppose a controller governs multiple microservices and a microservice should be controlled by only a controller. Hence, we define the variable $y_{n,c}^{i}$ as follows:

(1) $$y_{n,c}^{i}=\left\{\begin{array}{cc} 1, & \mathrm{if}\;\mathrm{microservice}\;i\;\mathrm{on}\;\mathrm{edge}\;\mathrm{server}\;n\\ \; & \mathrm{is}\;\mathrm{managed}\;\mathrm{by}\;\mathrm{controller}\;c\\ 0, & \mathrm{otherwise}. \end{array}\right.$$

If *n* = *c*, we have the controller manage microservices that are implemented on the same edge server as the controller. Assuming that each microservice can only be managed by one controller. We have the following:

(2) $$\sum_{c\in C}y_{n,c}^{i}=1,\forall i\in I,\;n\in N,$$

where *I* = {1, 2, 3, …, *i*} is the set of microservices.

Assume that some microservices are already placed on edge servers, given the on-demand nature of deployment. The computing capacity of the edge server *n* is denoted as $C_{n}$. We define the per-bit delivery delay between servers *n* and *m* as follows:

(3) $$t_{b(n,m)}=\frac{1}{c_{n,m}},$$

where $c_{n,m}$ is the channel capacity between *n* and *m*; according to Shannon’s theorem, we define $c_{n,m}$ as follows:

(4) $$c_{n,m}=W\log_{2}\left(1+\frac{P_{n}g_{0}\varepsilon_{\left(i,n\right)}d_{n,m}^{-\beta }}{E+\sigma^{2}}\right),$$

where *W* is the channel bandwidth between servers *n* and *m*, $P_{n}$ is the transmission power of the edge server *n*, $g_{0}$ refers to the transmission loss constant, and $\varepsilon_{\left(i,n\right)}$ and $d_{n,m}$ refer to the fading channel power gain and the distance from edge server *n* to edge server *m*. β represents the path loss exponent, *E* is the maximum received interference power, and $\sigma^{2}$ represents the Gaussian noise.

Additionally, a remote registry stores all container images. To enhance accessibility and reliability, the registry is typically hosted on a cloud platform. We define the download cost from the registry to edge server *n* as $Cost_{R,n}$, which is given as follows:

(5) $$Cost_{R,n}=\frac{Z_{l}}{B_{\left(R,n\right)}}+D_{\left(R,n\right)},$$

where $Z_{l}$ represents the size of layer *l* to be downloaded, $B_{\left(R,n\right)}$ is the bandwidth from the remote registry to edge server *n*, and $D_{\left(R,n\right)}$ is the propagation delay between them. It is important to note that the download cost varies across different edge servers. For an APP that needs to be implemented on the edge cloud, it is represented as APP = {*I*,*D*}, where *I* is the set of all microservices and *D* is the dependencies between the microservices. $d_{ij}\in D$ means that during the operation of microservice *i*, a total data size of $S_{ij}$ is transferred from microservice *i* to microservice *j* for interaction. In addition, as container images are layered, let *L* denote the set of all layers of microservices of this APP. Because different containers require different layers, a variable $\beta_{ij}\in \left\{0,1\right\}$ is defined. $\beta_{ij}=1$ means that microservice *i* contains layer *l* while $\beta_{ij}=0$ indicates the opposite meaning. Since a certain number of microservices are already placed on the edge cloud, a variable $\alpha_{ln}$ is employed to imply the placement of the shared layer. If server *n* has layer *l*, we have $\alpha_{ln}$ = 1. Otherwise, $\alpha_{ln}$ is set to 0.

## 5. Communication Overhead and Deployment Cost Calculation

Our solution for microservice deployment consists of three interconnected components. In Section 5, we calculate the system communication overhead and deployment cost. We then formulate a multi-objective optimization problem aimed at minimizing both metrics and transform it into a single-objective optimization problem, proving that the problem is NP-hard. In Section 6, we present the proposed RIME-ACPO algorithm designed to effectively solve the single-objective problem. In Section 7, we present the dynamic load balancing algorithm, which dynamically adjusts the microservice deployment based on the solution from the RIME-ACPO algorithm to further reduce the response time of user applications.

### 5.1. Modeling of Microservice Deployment

When a new application needs to be deployed on the edge cloud, the microservices it contains should be placed on the edge cloud. We define the binary variable $x_{in}$ to represent the placement decision of microservice *i*, and $x_{in}$ is given as follows:

(6) $$x_{in}=\left\{\begin{array}{cc} 1, & \mathrm{if}\;\mathrm{microservice}\;i\;\mathrm{is}\;\mathrm{deployed}\;\mathrm{on}\;\mathrm{edge}\;\mathrm{server}\;n\\ 0, & \mathrm{otherwise}. \end{array}\right.$$

To guarantee the integrity of microservice deployment, we have the following:

(7) $$x_{in}\in \left\{0,1\right\},\forall i\in I,\forall n\in N.$$

(8) $$\sum_{n\in N}x_{in}=1,\forall i\in I.$$

Let $P_{i}$ represent the computing resources required by microservice *i*. The computing resources consumed by all microservices deployed on an edge server must not surpass the servers’ computing capacity, which is denoted as follows:

(9) $$\sum_{i\in I}x_{in}P_{i}\leq C_{n},\forall_{n}\in N.$$

Additionally, when deploying a microservice, its associated container image must be downloaded to the edge cloud. Since container images are layered, not all layers of the image need to be pulled. Taking into account the layered structure of the microservice container image, a binary variable $z_{iln}$ is introduced to indicate whether layer *l* of microservice *i* has been placed on edge server *n*. If multiple microservices share the same layer, and at least one microservice has the shared layer deployed on server *n*, this layer does not need to be downloaded again. To download layer *l* from the remote registry to the edge server *n*, the following prerequisites are met: (1) the microservice that includes the layer ($\beta_{il}=1$) needs to be deployed to the server ($x_{in}=1$); (2) the layer does not exist on the server *n* ($\alpha_{ln}=0$). That is:

(10) $$\begin{array}{cc} z_{iln} & =x_{in}\beta_{il}\left(1-\alpha_{ln}\right)\left(1-max\left\{x_{jn}\beta_{jl}|\forall_{j}\in I,i\neq j\right\}\right),\;\forall n\in N,\forall i\in I. \end{array}$$

Therefore, to deploy the layers on the edge servers, the deployment cost is given as follows:

(11) $${Cost}_{dep}=\sum_{n\in N}\sum_{i\in I}\sum_{l\in L}{Z_{l}Cost}_{R,n}x_{in}.$$

The communication overhead of the system consists of two parts: (1) the overhead incurred by the communication between dependent microservices of the application. (2) The data delivery cost between microservices and the controllers that controls the microservices. The communication overhead arising from interactions between microservices is given as follows:

(12) $$T_{mic}=\sum_{n\in N}\sum_{m\in N}\sum_{i\in I}\sum_{j\in I}x_{in}x_{jm}S_{ij}t_{b(n,m)}.$$

Let $\lambda_{n}^{i}t_{b(n,m)}$ denote the overhead between microservice *i* on edge server *n* and the controller on edge server *m* that manages microservice *i*. The controller-to-microservice overhead is as follows:

(13) $$T_{mic,c}=\sum_{n\in N}\sum_{m\in N}\sum_{i\in I}x_{in}x_{jm}\rho_{c}\lambda_{n}^{i}t_{b(n,m)},$$

where $\rho_{c}$ represents the amount of data transferred between the microservice and the controller, $\lambda_{n}^{i}$ is the rate of requests of microservice *i* on server *n*, and $t_{b(n,m)}$ is the per bit delivery delay between servers *n* and *m*.

Therefore, the total communication overhead is given as follows:

(14) $$T_{all}=T_{mic}+T_{mic,c}.$$

The optimization objectives in our paper are twofold: (1) to minimize the system communication overhead presented in Formula (15), (2) to minimize the deployment cost presented in Formula (16):

(15) $$Min\left(T_{all}\right),$$

(16) $$Min\left({Cost}_{dep}\right).$$

We adopt the weighted method to consider these two objectives comprehensively and transform them into a single objective optimization problem aimed at minimizing the total cost, which is given as follows:

(17) $$\begin{array}{cc} P:\; & Min\left(\left(1-\omega \right)\;Cost_{dep}+\omega T_{all}\right)\\ \; & s.t.:\;(2),\;\left(7)–(10\right), \end{array}$$

where Formula (2) shows that each microservice can only be managed by one controller. Formula (7) defines the binary decision variable $x_{in}$, where $x_{in}$ = 1 indicates that the microservice is deployed on edge server *n*, and  $x_{in}$ = 0, otherwise. Formula (8) shows that each microservice can only be deployed to a single edge server. Formula (9) shows that the computing resources consumed by all microservices deployed on an edge server must not surpass the servers’ computing capacity. Formula (10) is the prerequisite for downloading the layer to the server.

### 5.2. Proof Analysis

Formula (17) is a discrete combination optimization problem. To prove that the proposed optimization problem is NP-hard, we use a reduction method [27]. That is, the microservice deployment problem is proved to be a special case of the Multi-dimensional Bin Packing Problem (MDBPP), which is a well-known NP-hard problem.

**Theorem** **1.** *The simplified microservice deployment problem proposed in this paper is an NP-hard problem.*

**Proof** **of** **Theorem** **1.** Let the bin in the MDBPP correspond to the server while the capacity of the bin corresponds to the capacity of the server. Items in MDBPP correspond to the microservices while the size of each item corresponds to the required computing resources of each microservice. In MDBPP, a fixed storage cost is assigned to each bin. That is, each time a bin is activated, a fixed resource consumption cost is incurred. In this way, the goal of minimizing the number of bins can be equivalently expressed as minimizing the total storage costs of the bins. In the microservice deployment problem, these fixed storage costs directly correspond to the deployment cost when microservices are deployed to the servers. We want to minimize the total cost, which consists of the communication overhead and the deployment cost. The deployment cost is assumed to be fixed. Further, since the controllers are deployed on servers, the interaction overhead between a microservice and a controller is simplified to the data delivery overhead between the microservice and microservices deployed on the edge server where the controller is placed.

Consequently, the system communication overhead in the optimization problem is reduced to the communication overhead between microservices, which, in the context of MDBPP, is equivalent to the communication overhead between items deployed in the bins in MDBPP. Hence, the original optimization problem *P* is converted into the following MDBPP:

(18) $$\begin{array}{cc} P^{\prime }:\; & Min\left(\left(1-\omega \right)\;{{Cost}^{\prime }}_{dep}+\omega {T^{\prime }}_{all}\right)\\ \; & s.t.:\;(2),\;\left(7)–(10\right), \end{array}$$

where ${{Cost}^{\prime }}_{dep}$ is the deployment cost and ${T^{\prime }}_{all}$ is the communication overhead between items.

The standard MDBPP is an NP-hard problem. Introducing communication costs between items allows the problem to be reduced to the NP-hard Quadratic Assignment Problem (QAP) [28]. Additionally, extra constraints increase the complexity of the problem. Therefore, the problem of deploying multiple items in bins to minimize the communication cost between items is an NP-hard problem. Through the above reduction, we demonstrate that the simplified microservice deployment problem is NP-hard. Hence, the proposed microservice deployment problem must also be NP-hard.    □

## 6. The RIME-ACPO Algorithm

Given the NP-hard nature of the problem, obtaining an optimal solution within polynomial time is computationally impractical. Therefore, to determine a suboptimal solution for this optimization, the RIME-ACPO algorithm is proposed. The RIME-ACPO algorithm combines the RIME algorithm [29] with an enhanced version of the CPO algorithm [30] proposed in this paper, termed as the Adaptive Crested Porcupine Optimizer (ACPO) algorithm.

The RIME algorithm is inspired by the natural formation process of rime ice. Soft rime facilitates a global search for potential solutions, while hard rime focuses on local refinement. Although RIME provides a powerful global search capability, it may be difficult to converge to an optimal solution when the parameters are poorly selected or the problem to be solved is complex. By simulating the intricate defensive behavior of the crowned porcupine, CPO is particularly good at handling local searches and detailed adjustments; it can further optimize the solution based on the search results provided by RIME. Combining these two algorithms can make full use of both the global search advantages of RIME and the local refinement capability of CPO, enabling a more effective approach to the global optimal solution and enhancing the solution accuracy. However, the Cyclic Population Reduction (CPR) method of the CPO, which is designed to maintain population diversity, confronts the challenge of stagnant solution quality across multiple consecutive iterations. To solve the above problem, we propose the ACPO algorithm, which can adaptively adjust the population size according to the quality of the solution during the optimization process.

### 6.1. The CPO Algorithm

CPO solves a complex optimization problem by simulating four defensive behaviors of Crowned Porcupine (CP): sight, sound, order, and physical attack. Sight and sound reflect the exploration stage of CPO while order and physical attack reflect the exploitation stage. The equation to simulate the first defense mechanism is as follows:

(19) $$\vec{x_{i}^{t\;+\;1}}=\vec{x_{i}^{t}}+\tau_{1}\times \left|2\times \tau_{2}\times \vec{x_{CP}^{t}}-\vec{y_{i}^{t}}\right|.$$

where $\vec{x_{i}^{t}}$ represents the position of the *i*-th individual in the current iteration; $\vec{x_{i}^{t\;+\;1}}$ represents the position of the *i*-th individual in the next iteration; $\vec{x_{CP}^{t}}$ is the best solution; $\vec{y_{i}^{t}}$ is a vector that is generated between the current CP and a randomly selected CP from the population, representing the position of a predator at iteration *t*; $\tau_{1}$ is a normally distributed random number; $\tau_{2}$ is a random value within [0,1].

The second defense approach is given as follows:

(20) $$\vec{x_{i}^{t+1}}=\left(1-\vec{U_{1}}\right)\times \vec{x_{i}^{t}}+\vec{U_{1}}\times \left(\vec{y}+\tau_{3}\times \left(\vec{x_{r_{1}}^{t}}-\vec{x_{r_{2}}^{t}}\right)\right),$$

where $\vec{U_{1}}$ is a randomly generated value used to regulate the weight between the current position and the new position, $\tau_{3}$ is a random value within [0,1], $\vec{x_{r_{1}}^{t}}$ and $\vec{x_{r_{2}}^{t}}$ are two different individuals randomly selected from the population.

The equation to simulate the third defense mechanism is as follows:

(21) $$\begin{array}{cc} \vec{x_{i}^{t+1}}= & \left(1-\vec{U_{1}}\right)\times \vec{x_{i}^{t}}+\vec{U_{1}}\times \left(\vec{x_{r_{1}}^{t}}+S_{i}^{t}\times \left(\vec{x_{r_{2}}^{t}}-\vec{x_{r_{3}}^{t}}\right)\right.-\tau_{3}\times \vec{\delta }\times \gamma_{t}\times S_{i}^{t}), \end{array}$$

where $\vec{\delta }$ is used to control the search direction, $\gamma_{t}$ is the defense coefficient, and $S_{i}^{t}$ is the odor diffusion coefficient.

The fourth defense mechanism is denoted as follows:

(22) $$\begin{array}{cc} \vec{x_{i}^{t+1}} & =\vec{x_{CP}^{t}}+\left(\alpha \left(1-\tau_{4}\right)+\tau_{4}\right)\times \left(\delta \times \vec{x_{CP}^{t}}-\vec{x_{i}^{t}}\right)-\tau_{5}\times \delta \times \gamma_{t}\times \vec{F_{i}^{t}}, \end{array}$$

where α represents the convergence rate coefficient and $\vec{F_{i}^{t}}$ denotes the average force exerted by the CP of the *i*-th predator.

Furthermore, the CPR method is introduced to maintain population diversity while accelerating the convergence of the CPO. The mathematical model for cyclically reducing the population size is defined as follows:

(23) $$N=N_{min}+\left(N^{\prime }-N_{min}\right)\times \left(1-\left(\frac{t\%\frac{T_{max}}{T}}{\frac{T_{max}}{T}}\right)\right),$$

where *T* represents a variable that defines the number of iterations, *t* is the current evaluation of the function, $T_{max}$ is the total number of function evaluations allowed, $N^{\prime }$ is the number of candidate solutions, and $N_{min}$ is the minimum number of individuals in the newly created population, ensuring that the population size cannot fall below $N_{min}$.

By performing the above-mentioned approaches, CPO can not only efficiently search the solution space, but also adaptively adjust the search strategies to cope with the changing environment.

### 6.2. The RIME-ACPO  Algorithm

In our proposed ACPO algorithm, to dynamically adjust the solution based on the historical improvement of the solution, an adaptive factor μ(t) is introduced, where *t* represents the latest iteration period. We have the following:

(24) $$\mu \left(t\right)=\exp\left(-\gamma \cdot \Delta f(t)\right),$$

where Δf(t)=|f(t)−f(t−1)| indicates the difference in objective function value between the latest solution and the previous solution, and γ is the sensitivity parameter of the adjustment factor, designed to control the reaction speed and the amplitude of μ(t).

Formula (23) is then modified as follows:

(25) $$\begin{array}{c} N=\left(N_{\min}+\left(N^{\prime }-N_{\min}\right)\times \left(1-\left(\frac{t\%\frac{T_{max}}{T}}{\frac{T_{max}}{T}}\right)\right)\right)\times \mu \left(t\right). \end{array}$$

In each iteration, we calculate Δf(t) and μ(t). Then, we apply Formula (25) to adjust the population size. As μ(t) is adjusted based on the improvement of the solution, compared with the original CPR method, the population size becomes more flexible and better aligned with the requirements of the search process. Note that the adjusted population size is always within $\left[N_{min},N^{\prime }\right]$, with the aim of avoiding the population size being too large or too small.

Algorithm 1 presents the pseudo-code of the proposed RIME-ACPO algorithm, where *t* indicates the current number of iterations and *T* is the maximum number of iterations. Line 3 describes that the fitness value of the current solution is first calculated in each iteration. Lines 4–13 use RIME’s soft-rime search and hard-rime puncture to explore the fitness values of the updated and pre-updated solutions, where $\sqrt{\frac{t}{T}}$ normalizes the current progress and $F^{normr}\left(S_{i}\right)$ represents the normalized fitness value of the *i*-th individual. If the updated fitness value surpasses the pre-updated fitness value, the two solutions, along with their corresponding fitness values, are replaced. The updated population is then returned. Line 12 represents the final RIME solution. Lines 14 and 15 denote that the adaptive factor μ(t) is calculated and the population size is adjusted. Lines 16–22 express that the exploration stage is performed if $rand<rand_{0}$, where rand and $rand_{0}$ are two random numbers within [0,1]. If $rand_{1}<rand_{2}$, the first strategy is implemented; otherwise, the second strategy is implemented. Lines 23–29 express that the exploitation stage is carried out if $rand>rand_{0}$. If $rand_{3}<T_{f}$, where $T_{f}$ is a predetermined constant value between 0 and 1, the third strategy is implemented; otherwise, the fourth strategy is implemented. In Lines 30–33, new potential solutions are continually introduced, and the fitness value is recalculated to identify a solution with a deployment that outperforms the previous placement solution. Line 37 returns the final optimal solution. The main symbols in this paper and their corresponding meanings are summarized in Table 1.
**Algorithm 1** RIME-ACPO algorithm.**Input:** the initialization of the microservice deployment $X_{in}$, the number of microservices *I*, the number of servers *N*, the number of layers *L*, the computing resource $C_{n}$, the computing resource required by microservice $P_{i}$, $\beta_{il}$, $\alpha_{ln}$, $Z_{l}$, $S_{ij}$, $\lambda_{n}^{i}$, $\rho_{c}$, layer sharing rate**Output:** $X_{in}$  1:**for** i∈I,n∈N and l∈L **do**  2:      **while** t<T **do**  3:          Calculate the fitness by Equation (17)  4:          **if** random $value<\left(\sqrt{\frac{t}{T}}\right)$ **then**  5:              Update RIME agent by soft-rime search  6:          **end if**  7:          **if** random $value<F^{normr}\left(S_{i}\right)$ **then**  8:              Update RIME agent by hard-rime puncture  9:          **end if**10:          **if** new fitness<old fitness **then**11:              Select the optimal solution and replace the suboptimal solution12:              **return** updated $X_{in}$13:          **end if**14:          Update μ(t) by Equation (24)15:          Adjust population size by Equation (25)16:          **if** $rand<rand_{0}$ **then**17:              Generate three random numbers, $rand_{1}$, $rand_{2}$, and $rand_{3}$18:              **if** $rand_{1}<rand_{2}$ **then**19:                  Apply Equation (19)20:              **else**21:                  Apply Equation (20)22:              **end if**23:          **else**24:              **if** $rand_{3}<T_{f}$ **then**25:                  Apply Equation (21)26:              **else**27:                  Apply Equation (22)28:              **end if**29:          **end if**30:          **if** $F\left(\vec{x_{i}^{t\;+\;1}}\right)>F\left(\vec{x_{i}^{t}}\right)$ **then**31:              $\vec{x_{i}^{t\;+\;1}}=\;\vec{x_{i}^{t}}$32:              **return** updated $X_{in}$33:           **end if**34:           t=t+135:      **end while**36:**end for**37:**return** best solution $X_{in}$

Our proposed algorithm builds upon the CPO algorithm by integrating the RIME optimization technique to enable efficient microservice deployment. The algorithm employs *T* outer iterations, with each iteration sequentially processing parameters *I*, *N*, and *L* in an inner loop structure, where *N* is the number of edge servers, *I* is the number of microservices, and *L* is the number of layers. It also consists of several key components, each with its corresponding complexity. The complexity of the fitness function calculation of RIME is O(f), where *f* is the fitness value of Equation (17). The complexity of RIME’s soft-rime search and hard-rime puncture mechanism is O(n·d), where *n* is the number of individual variables and *d* is the perturbation range of each variable. The complexity of the greedy selection mechanism is O(nlogn). The complexity of ACPO is $O(\frac{N_{\max}\;+\;N_{\min}}{2}\cdot f)$, which depends on the number of candidate solutions N, and fitness calculation where $N_{max}$ is the maximum population size module while $N_{min}$ is the minimum population size module. A comprehensive analysis shows that the total complexity of the algorithm is $O\left(T\cdot I\cdot N\cdot L\cdot \left(f+n\cdot d+\log n+\frac{N_{\max}\;+\;N_{\min}}{2}\cdot f\right)\right)$.

## 7. Dynamic Load Balancing

Load balancing is crucial for ensuring the high availability, scalability, and performance of applications and services. Moreover, load balancing is of vital importance to the performance of user services [7]. Hence, it is critical to consider load balancing during microservices placement. When a server is overloaded, microservice migration is required. For each edge server, the system continuously monitors the microservices placed on it and calculates its resource usage. For edge server *n*, the resource usage rate is as follows:

(26) $$U_{n}=\frac{\sum_{i\in APP}\sum_{l\in i}P_{i}x_{in}}{C_{n}}.$$

The variance of resource usage rate of all the edge servers is represented as follows:

(27) $$V_{u}=\frac{1}{\left|N\right|}\sum_{n\;=\;1}^{\left|N\right|}{\left(U_{n}-\overset{-}{U}\right)}^{2},$$

where $U_{n}$ represents the resource utilization rate of server *n*, and $\overset{-}{U}$ represents the average resource usage rate. The smaller the value of $V_{u}$, the more balanced the load distribution.

We define a threshold η. If $V_{u}>\eta$, the edge cloud’s load is unbalanced. In this condition, we select one of the microservices on the edge server with the largest resource load for migration. After migrating a microservice, the value of $V_{u}$ is recalculated. If $V_{u}>\eta$ still holds, the microservice migration process continues. The dynamic load balancing algorithm is shown in Algorithm 2.
**Algorithm 2** Dynamic load balancing.**Input:** *U*, *V*, $T_{all}$, ${Cost}_{dep}$, the weight of $T_{all}$ σ, the weight of ${Cost}_{dep}$ φ, the weight of *V* (1−σ−φ), and the threshold η**Output:** the index index of the microservice  1:Sort in descending order of *U*  2:$n_{1}$ is the node with the largest load; $n_{2}$ is the node with the lightest load  3:tV=0  4:$tT_{all}=0$  5:$t{Cost}_{dep}=0$  6:index=0  7:**if** V>η **then**  8:    ${Score}_{u}=\;+\infty$  9:    **for** *i* in $n_{1}$ **do**10:          tV= calculate the variance11:          $tT_{all}=$ calculate $T_{all}$12:          $t{Cost}_{dep}=$ calculate ${Cost}_{dep}$13:          ${Score}_{usage}=\left(1-\sigma -\varphi \right)\left(tV-V\right)$14:          ${Score}_{com}=\sigma \left(tT_{all}-T_{all}\right)$15:          ${Score}_{dep}=\varphi \left(t{Cost}_{dep}-{Cost}_{dep}\right)$16:          **if** ${Score}_{usage}+{Score}_{com}+{Score}_{dep}\;<\;{Score}_{u}$ **then**17:            $index=n_{2}$18:            ${Score}_{u}=\;{Score}_{usage}+{Score}_{com}+{Score}_{dep}$19:        **end if**20:    **end for**21:**end if**22:**return** index

It should be noted that the algorithm only migrates one microservice at a time. The algorithm evaluates the impact of relocating each microservice from the edge server with the highest resource utilization to a target node, focusing on how this transfer affects the total cost. It then selects the microservice with the least impact on the total cost for migration. Lines 1 and 2 indicate that the resource utilization rates of all servers are sorted in descending order. Lines 3–6 initialize the current variance tV, communication overhead $tT_{all}$, and deployment cost $t{Cost}_{dep}$, and index is used to record the target edge server number that is finally decided to migrate. Lines 7–9 specify that when the variance exceeds the threshold, the algorithm traverses all microservices on the server with the highest resource utilization and initializes the current best score to +∞. Lines 10–21 denote the calculation of the scores of all microservices on this edge server. To minimize the total cost variation during microservice relocation, the algorithm determines the optimal migration node by calculating three metrics: ${Score}_{usage}$, ${Score}_{com}$, and ${Score}_{dep}$. Finally, the index of the microservice selected for transfer is returned.

The complexity of sorting the edge servers by their network loads in the initial stage is O(NlogN). The complexity of traversing the microservices on the server with the highest load to calculate the load variance, communication latency, and deployment cost of each microservice after migration, and scoring them to select the optimal migration plan, is O(M). Therefore, the complexity of Algorithm 2 is O(NlogN+M).

## 8. Performance Evaluation

### 8.1. Dataset and Experimental Setup

To analyze the performance of our algorithm, we used the EUA dataset [31], which features 125 edge servers positioned across different sites in the central business district of Melbourne, Australia. We randomly selected 15 edge servers for simulation. Furthermore, to ensure the credibility of our experimental results, we employed the Alibaba Cluster Tracer [32], which offers comprehensive data of 20,000 microservices across more than 10 clusters. For our experiment, we selected 10 microservices that constitute an application for evaluation. These microservices consist of a total of 30 layers. Since it is challenging to obtain hierarchical structure information of microservices from open tracing services, we manually configured 6 read-only layers to simulate hierarchical sharing. Specifically, 6 layers were shared among 10 microservices. The default parameters of the experiment are shown in Table 2. A total of 15 edge servers simulated a medium-scale edge computing environment, balancing complexity and computational cost. A total of 10 microservices, which is 1–15 MB, modeled real-world deployment needs. Then, 2 controllers reduced communication bottlenecks and improved resource allocation, with tests on controller count impact. A 20% layer sharing rate reflects real container image sharing, with experiments on varying rates. The layer size $Z_{l}$, which was 1–15 MB, suits lightweight edge microservices. $Cost_{R,n}$ was 5–15 ms for downloading layers depends on the layer size (1–15 MB), bandwidth (500 Mbps–2 Gbps), and propagation delay (1–9 ms), according to Formula (5). $P_{i}$, which was 10–25 MHz, reflects microservice computing needs. $C_{n}$, which was 1200–1600 MHz, matched the edge server capacity according to the literature [6]. $S_{ij}$, which was 2–8 MB, modeled microservice data exchange. $\rho_{c}$, which was 1–5 MB, reflected controller–microservice communication, according to the literature [15]. $\lambda_{in}$, which was 5–10 requests/s, balanced responsiveness and resource use. ω, which was 0.6, balanced communication and deployment costs, adjustable for optimization.

In real cloud-native computing, the number of microservices is typically dynamically scaled based on the volume of user requests. When analyzing user response time, we adopt a dynamic scaling mechanism to simulate the edge cloud environment as realistically as possible. For each microservice, its corresponding usage is periodically estimated. If the average value exceeds the threshold, the number of instances of this microservice will be increased to prevent an increase in user response time. Based on References [33,34], the dynamic scaling threshold was set to 0.8. We submitted multiple user requests to the system and defined the user response time for each request as the sum of three components: the submission delay of the user request, the communication time between the microservices that constitute the application, and the pull time of the microservices. For simplicity, we assumed that the user request submission delay ranged between 100 and 200 ms in the experiment.

The comparison algorithms are as follows:

1. The LA-MPRS algorithm [35]. It reduces the deployment cost of layers by taking advantage of the layer sharing feature of container images. The shared layers are placed together to reduce the deployment cost of the microservices.
2. The HCM algorithm [36]. It leverages the inter-dependencies among microservices to co-locate frequently interacted microservices on the same servers, thereby minimizing the communication overhead during runtime.
3. The RIME algorithm. This algorithm finds the optimal solution by constructing the soft-rime search strategy and the hard-rime puncture method.
4. The RIME-CPO algorithm. This algorithm combines RIME with the CPO. RIME-CPO optimizes the solution using the CPO algorithm based on the search results provided by RIME.
5. The DQN algorithm (Deep Q-Network) [37]. This algorithm approximates the Q-value function using deep neural networks, enabling agents to learn optimal policies from high-dimensional state spaces through trial-and-error interactions with the environment.

### 8.2. Evaluation Metrics

The evaluation metrics are as follows:

1. User response time: It is the time taken from when a user submits a request to when the user receives a response. It is an important metric in edge computing because it directly impacts the user experience.
2. The total cost: The total cost consists of system communication overhead and deployment cost. By accounting for these two critical factors, it provides an accurate evaluation of the system’s overall performance and cost-effectiveness in edge computing environments.
3. System communication overhead: It is composed of the communication overhead between microservices and the interaction overhead between microservices and controllers, referring to the time spent on data transmission and communication within the system.
4. Microservice deployment cost: It refers to the cost associated with deploying microservices from the remote registry to edge servers.

### 8.3. Results Analysis

#### 8.3.1. User Response Time of the Algorithms

Table 3 presents the average user response times for different algorithms, demonstrating that our RIME-ACPO algorithm achieves the lowest user response time. Specifically, it outperforms LA-MPRS by 3.9 s and RIME-CPO by 0.9 s. This improvement is primarily attributed to the fact that, unlike the LA-MPRS and HCM algorithms, our proposed approach accounts for both system communication overhead and microservice deployment cost during the optimization process, effectively reducing the user response time. Furthermore, compared to the RIME and RIME-CPO algorithms, RIME-ACPO adaptively adjusts the population size, making it more efficient at finding the optimal solution, thus achieving a lower user response time. The user response time of our algorithm is slightly lower than that of the DQN algorithm, primarily due to the fact that our algorithm enhances the solution’s precision through local search while ensuring global search capabilities. This also demonstrates the greater stability of RIME-ACPO when dealing with large-scale data and complex constraints. Further, the training time of RIME-ACPO is less than 10 s, which is lower than the 32 s required by DQN. In edge computing environments, both network conditions and computational resources are subject to dynamic fluctuations. Traditional deep reinforcement learning methods typically necessitate frequent model retraining and updates to adapt to such variability. This continuous updating process often results in prolonged training durations and substantial computational overhead, particularly in large-scale edge computing environments. On the other hand, RIME-ACPO combines the advantages of RIME and ACPO, ensuring global search capability while achieving faster convergence and obtaining superior solutions within a limited computation time.

**Figure 5:**
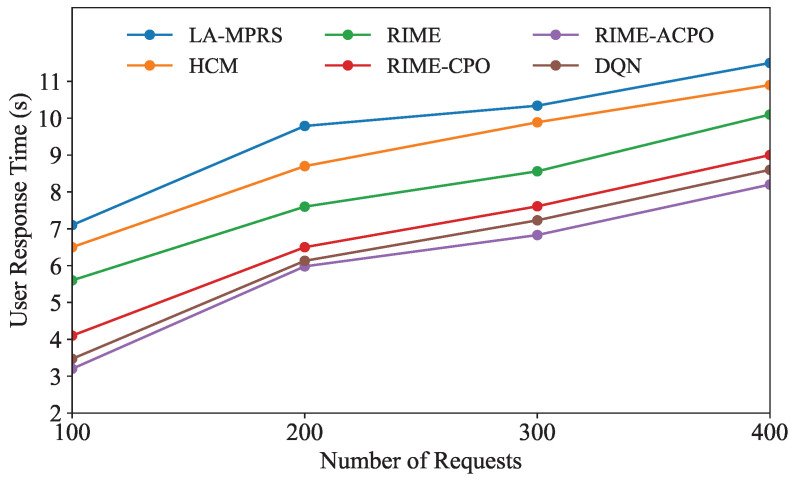
User response time under different numbers of requests.

Figure 5 illustrates how the user response time of each algorithm changes with the number of user requests. As shown in Figure 5, the user response times of all algorithms exhibit an increasing trend as the number of user requests grows. This is because the rise in the number of user requests leads to increased competition for a fixed amount of system resources, resulting in longer user response times of user applications. As the number of requests varies, the average user response time of our algorithm consistently remains lower than that of the other five comparison algorithms, proving the superiority of RIME-ACPO.

#### 8.3.2. Comparison Under Different Layer Sharing Rates

**Figure 6:**
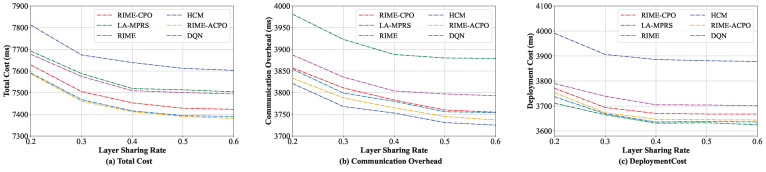
Performance under various layer sharing rates.

In this group of experiments, the performance of these algorithms in comparison under different layer sharing rates was evaluated. We progressively increased the layer sharing rate from 20% to 60%, with the experimental results presented in Figure 6. As illustrated in Figure 6a, the total cost of RIME-ACPO is lower than the other five algorithms. The performance of our algorithm is better than the RIME and RIME-CPO algorithms. The main reason is that our algorithm employs the improved CPR, which not only further optimizes the optimal solution obtained by RIME but also adaptively adjusts the population compared to CPR and fine-tunes the global solution. In addition, our algorithm performs better than DQN in the total cost. The main reason is that RIME-ACPO directly eliminates invalid solutions through dynamic constraint handling, outperforming DQN’s reward-shaped optimization in microservice deployment. Its dual-population approach preserves diversity, reducing DQN’s typical local optima and catastrophic forgetting issues. LA-MPRS outperforms HCM, with HCM exhibiting the highest total cost among the algorithms in comparison. To acquire a more comprehensive understanding of the efficiency of the proposed algorithm, the communication overheads and the deployment costs of these six algorithms are further detailed in Figure 6b and Figure 6c, respectively. As illustrated in Figure 6b, the LA-MPRS algorithm incurs the highest communication overhead, whereas HCM demonstrates superior performance compared to the other five algorithms in terms of communication overhead. On the contrary, for the deployment cost, LA-MPRS performs the best. This is because the primary objective of the LA-MPRS algorithm is to maximize the benefits of layer sharing, without considering the communication overhead between microservices, which leads to increased communication overhead between microservices. Furthermore, the HCM algorithm focuses on deploying frequently communicating microservices on the same edge server, completely overlooking the reduction in deployment cost, which leads to the highest deployment cost.

#### 8.3.3. Comparison When Varying Computing Resource Capacity

**Figure 7:**
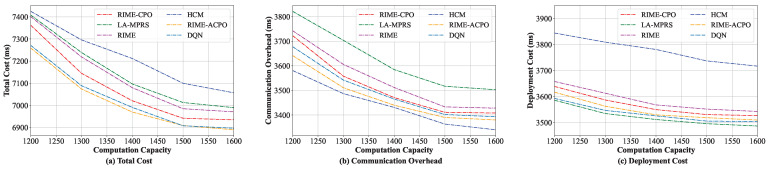
Performance under various computing resources.

In this group of experiments, the performance of different algorithms when varying the edge server’s computing resource capacity was evaluated. The computing capacity was increased from 1200 MHz to 1600 MHz. As shown in Figure 7a, the total costs of the six algorithms are all greatly affected by the computing capacity. The main reason is that a higher computing capacity enables more microservices to be deployed on the edge servers. This not only enhances the likelihood of layer sharing when microservices are co-located but also significantly minimizes communication overhead by centrally deploying frequently interacting microservices on the same server. As computing resource capacity varies, our algorithm consistently achieves the lowest total cost among the six algorithms, demonstrating its clear superiority. The main reasons are threefold: at first, compared with the HCM and LA-MPRS algorithms, our algorithm considers the communication overhead and the deployment cost minimization when placing microservices. Secondly, compared with the RIME and RIME-CPO algorithms, RIME-ACPO further enhances solution quality and accuracy through fine adjustments. Finally, RIME-ACPO outperforms DQN in microservice deployment. This may be because RIME-ACPO dynamically prunes invalid solutions through constraint handling, eliminating DQN’s reward-shaping reliance. Furthermore, its dual-population approach maintains solution diversity, thereby reducing the risk of being stuck in local optima and mitigating catastrophic forgetting.

Figure 7b,c illustrate the changes in communication overhead and deployment cost concerning the computation capacity, respectively. Figure 7b shows that as the computing resource capacity increases, the communication overheads of the six algorithms decrease and eventually tend to be stable. The HCM algorithm has the lowest communication overhead among the six algorithms. Figure 7c shows that as the computing resource capacity increases, the deployment costs of the six algorithms all gradually reduce. Since LA-MPRS considers deploying all microservices with layer sharing on the same server to reduce the image downloading cost, it incurs the lowest deployment cost.

#### 8.3.4. Comparison When Varying the Number of Controllers

**Figure 8:**
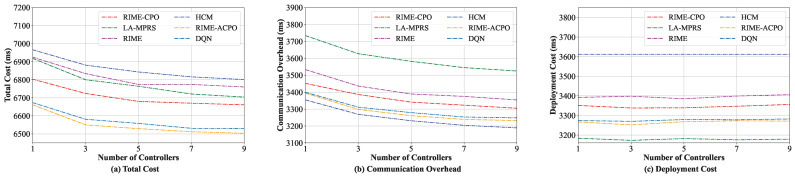
Performance under various numbers of controllers.

We also evaluated the effect of different numbers of controllers on the performance of various algorithms. The number of controllers was increased from 1 to 9. To address the challenges of microservice deployment in a large-scale edge cloud, the number of servers in this experiment was fixed at 50. As shown in Figure 8a, the total costs of all these algorithms decrease as the number of controllers increases. This is primarily because when multiple controllers exist in the system, the microservice can choose to communicate with the controller nearest to it, thus significantly reducing the microservices to the controller interaction cost. Hence, the total costs are reduced. Since the introduction of multiple controllers has little impact on the deployment cost, as shown in Figure 8c, the deployment cost does not change significantly. From Figure 8, we can see that our algorithm can achieve a lower total cost than other algorithms. Note that as the number of controllers increases, the interaction overhead between them grows. Therefore, the number of controllers should be carefully controlled and determined based on the scale of the edge cloud.

#### 8.3.5. The Value of ω on the Performance of RIME, RIME-CPO, and RIME-ACPO

**Figure 9:**
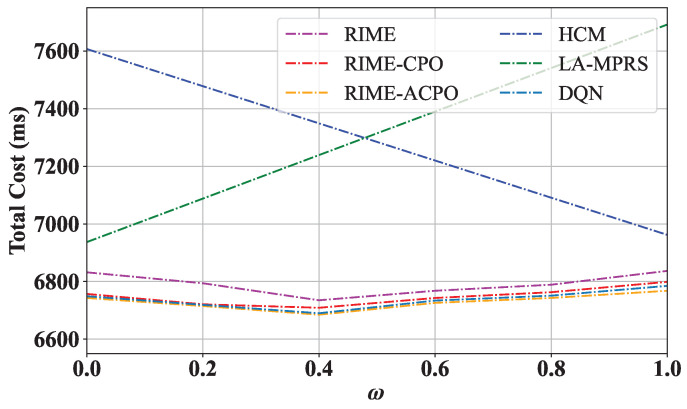
Total cost vs. different values of ω.

To verify the effect of the weight coefficient ω on the performance of RIME-ACPO, we changed ω from 0 to 1, and the results are shown in Figure 9. The HCM and LA-MPRS algorithms consistently incur higher total costs than the other four algorithms across varying ω values. For the RIME, RIME-CPO, RIME-ACPO, and DQN algorithms, their total costs first decrease and then increase with the increase in ω. When ω is large, the system prioritizes minimizing system communication overhead through decentralized microservice deployment, but this may increase deployment cost. Conversely, with smaller ω values, the system prioritizes reducing the deployment cost, resulting in the clustered deployment of microservices with shared layers at the expense of increased system communication overhead. The optimal balance occurs at ω=0.4, achieving the lowest total cost by equally weighing both factors. The adaptive population adjustment of ACPO not only maintains the periodic adjustment characteristics of CPR but also adds the ability to dynamically adjust according to real-time feedback during the optimization process, effectively improving the quality of the solution and the adaptability of the algorithm. Hence, the total cost of RIME-ACPO is lower than that of other algorithms. Figure 9 also demonstrates that the proposed algorithm has strong adaptability. For applications requiring a long operational time, minimizing communication overhead becomes a primary concern. Therefore, the weight ω should be set to a larger value.

#### 8.3.6. Algorithm Performance Under Different Network Scales

**Figure 10:**
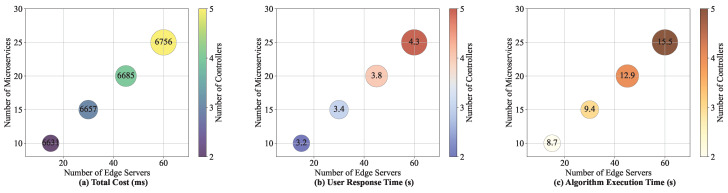
The changes in total cost and user response time at different scales.

We analyze the changes in user response time and total cost of these algorithms under different network scales, and the experimental results are shown in Table 4 and Figure 10. In the experiment, we gradually increased the number of edge servers and microservices. As the number of edge servers and microservices grows, the number of controllers is increased to minimize the communication latency between the controllers and microservices. It can be seen from Table 4 and Figure 10 that as the network scale increases, the user response time of each algorithm increases, and the total cost also shows an increasing trend. This is because the larger the network scale, the exponential growth of communication links between microservices and the coordination overhead between controllers also increases, and the microservice deployment cost increases, resulting in an increase in the overall response time and cost of the system. The total cost and user response time of the RIME-ACPO algorithm are better than those of the comparison algorithms under the change in network scale. When scaling the network from 15 to 60 nodes, as shown from Table 4 and Figure 10, the execution time of RIME-ACPO increases from 8.7 s to 15.5 s (a 78% rise), while the user response time only grows from 3.2 s to 4.3 s (a 34% increase). These results demonstrate that RIME-ACPO effectively maintains latency performance while introducing manageable computational overhead, achieving an optimal trade-off between computational cost and performance optimization.

#### 8.3.7. Dynamic Load Balancing

**Figure 11:**
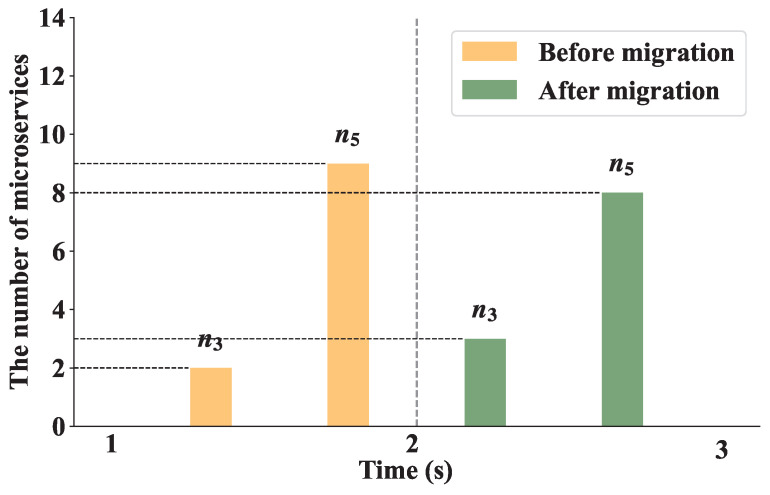
The first migration process.

**Figure 12:**
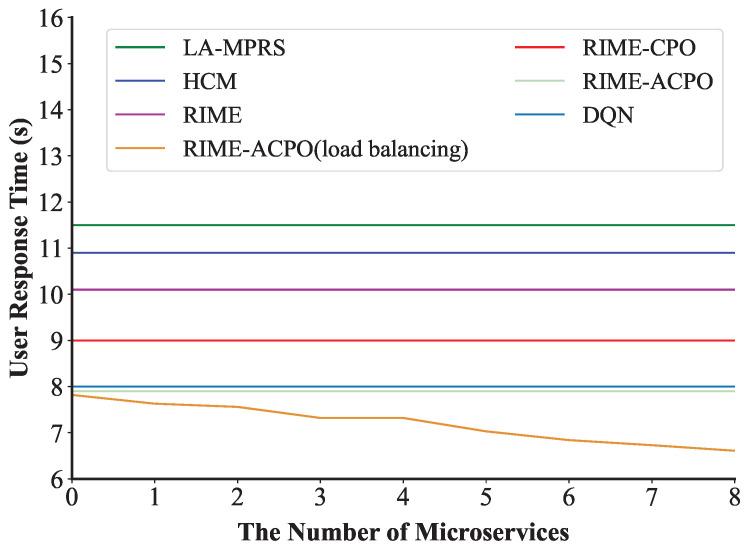
The change in user response time during the load balancing process.

**Figure 13:**
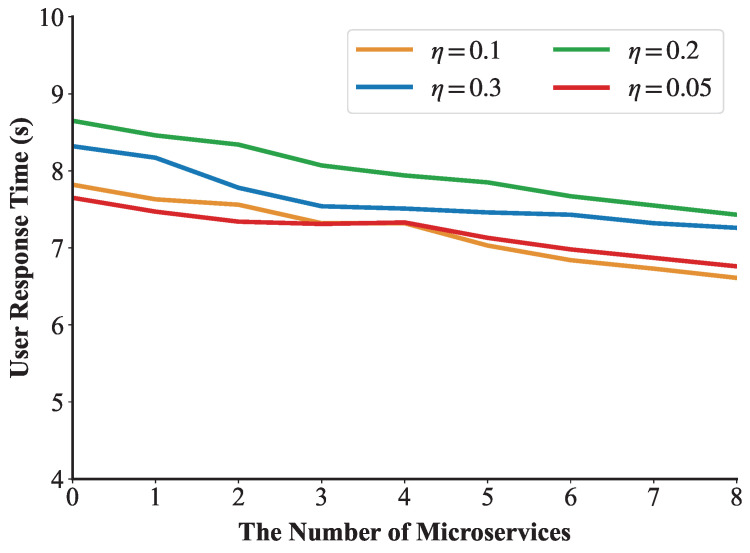
The change in user response time under different η values.

This experiment verified the effect of the load balancing algorithm when the number of user requests was 400. We set the threshold η as 0.1 to trigger the dynamic load balancing algorithm. Figure 11 shows the first execution process of the load balancing algorithm in the experiment. We can see that a microservice on edge $n_{5}$ is migrated to $n_{3}$ at 2 s to balance the network load. After the algorithm is executed, the number of microservices on $n_{3}$ increases from 2 to 3, and the number of microservices on $n_{5}$ decreases from 9 to 8. Figure 12 demonstrates that under a request rate of 400 requests per second, the RIME-ACPO algorithm achieves a lower user response time compared to other benchmark algorithms. When implementing our proposed dynamic load balancing algorithm based on the RIME-ACPO, the user response time progressively decreased as the number of migrated microservices increased. These results validate the effectiveness of our dynamic load balancing algorithm in reducing user response time. Figure 13 illustrates the variation in user response time with respect to the number of migrated microservices under different threshold values. It can be observed that, under the same conditions, when the number of algorithm executions does not exceed four, the algorithm achieves the best performance when η is 0.05. However, when the number of executions increases, the algorithm performs better with a threshold value of 0.1 compared to other values. The main reason is that if the threshold is set too large, some edge servers may incur resource competition due to excessive load, and the response time of the user request increases. Conversely, a threshold that is too low results in frequent migrations of resources on edge servers before they are fully utilized, leading to a higher system overhead and a prolonged user response time.

### 8.4. Summary and Analysis of Experimental Results

To comprehensively evaluate the performance of the proposed RIME-ACPO algorithm and dynamic load balancing algorithm, we conducted extensive experiments under various edge computing scenarios and compared our mechanism against some representative baselines. The results demonstrated that RIME-ACPO consistently outperformed the baselines across all evaluation metrics, including user response time and total cost. In particular, RIME-ACPO achieved up to 3.03% reduction in user response time, a 0.33% decrease in total cost, compared to the strongest baseline. Further, as the dynamic load balancing algorithm ran, the user response time continued to decrease. These improvements are largely attributed to the hybrid optimization mechanism combining RIME and ACPO, as well as the dynamic load balancing strategy, which adapts to runtime resource variations. Furthermore, the scalability experiments showed that the algorithm maintained a stable convergence behavior as the number of edge nodes and microservices increased, demonstrating its suitability for large-scale edge-native deployments.

Overall, the proposed mechanism provides a robust and efficient solution for latency-sensitive microservices.

## 9. Conclusions

In this paper, to decrease the user application response time, we propose a microservice deployment mechanism considering both the deployment cost and the communication overhead generated by microservices’ interactions and microservices-to-controller interactions under the multi-controller structure. Our goal was to optimize user response time by reducing both the system communication latency and the microservice deployment cost. Multiple controllers were integrated into the edge cloud to reduce the data transfer latency between microservices and controllers. During the microservice deployment process, we established an optimization problem and then proposed the RIME-ACPO algorithm to obtain the suboptimal solution. Further, we present a dynamic load balancing algorithm, which dynamically adjusts the microservices’ deployment by monitoring the resource occupancy of edge servers in a timely manner. Owing to the dynamic load balancing algorithm, load balancing in the edge cloud is ensured, effectively preventing a decline in service execution performance. A large number of experimental results show that the proposed algorithms are able to largely reduce the user response time and the total cost in comparison with other algorithms, demonstrating the effectiveness of the microservice deployment mechanism of this paper in enhancing user experience. We assume that the number of controllers is fixed. However, after the controllers are introduced, the controllers need to interact with each other, which causes the interaction overhead between controllers. This is our limitation. In the future, we will focus on addressing the optimization of controller quantity and placement. Moreover, employing artificial intelligence strategies such as deep reinforcement learning to reduce user response time and enhance system performance will be a key research direction. 

## Figures and Tables

**Table 1:** Main symbols table.

Symbol	Explanation	Symbol	Explanation	Symbol	Explanation
$Z_{l}$	the size of layer *l*	${Cost}_{R,n}$	the download cost from the registry to edge server *n*	$x_{in}$	a binary variable indicating whether microservice *i* is deployed on server *n*
$S_{ij}$	the total data size is transferred from microservice *i* to *j*	$t_{b(n,m)}$	the per-bit delivery delay between severs *n* and *m*	$\rho_{c}$	the amount of data transferred between the microservice and the controller
$\lambda_{n}^{i}$	the rate of requests of microservice *i* on server *n*	$\beta_{il}$	whether microservice *i* contains layer *l*	$\alpha_{ln}$	whether the shared layer *l* is deployed on server *n*
$Cost_{dep}$	the download cost	$T_{mic}$	the communication overhead between microservices	$T_{mic,c}$	the communication overhead between microservices and controllers
$T_{all}$	the total communication overhead	$U_{n}$	the resource usage rate	$V_{u}$	the variance of resource usage rate

**Table 2:** Default experimental parameters.

Parameter	Size
Number of edge servers	15
Number of microservices	10
Number of controllers	2
Layer sharing rate	20%
$Z_{l}$	1–15 MB
${Cost}_{R,n}$	5–15 ms
$P_{i}$	10–25 MHz
$C_{n}$	1200–1600 MHz
$S_{ij}$	2–8 MB
$\rho_{c}$	1–5 MB
$\lambda_{n}^{i}$	5–10 times/s
ω	0.6

**Table 3:** User response time of different algorithms.

Number of Requests	100
LA-MPRS	7.1 s
HCM	6.5 s
RIME	5.6 s
RIME-CPO	4.1 s
DQN	3.3 s
RIME-ACPO	3.2 s

**Table 4:** Comparison of methods with different resource configurations.

Method	Number of Edge Servers	Number of Microservices	Number of Controllers	Total Cost (ms)	User Response Time (s)
LA-MPRS	15	10	2	6863	7.1
	30	15	3	6882	7.3
	45	20	4	6923	7.8
	60	25	5	6944	8.2
HCM	15	10	2	6942	6.5
	30	15	3	6972	6.6
	45	20	4	7018	6.9
	60	25	5	7121	7.3
RIME	15	10	2	6887	5.6
	30	15	3	6889	5.8
	45	20	4	6912	6.3
	60	25	5	6920	6.6
RIME-CPO	15	10	2	6743	4.1
	30	15	3	6769	4.2
	45	20	4	6794	4.5
	60	25	5	6811	5.7
DQN	15	10	2	6653	3.3
	30	15	3	6668	3.5
	45	20	4	6705	4.0
	60	25	5	6784	4.5
RIME-ACPO	15	10	2	6631	3.2
	30	15	3	6657	3.4
	45	20	4	6685	3.8
	60	25	5	6756	4.3

## Data Availability

The data used for this study are available upon request from the corresponding author.

## Abbreviations

The following abbreviations are used in this manuscript:

RIME	RIME optimization algorithm
CPO	Crested Porcupine Optimizer
ACPO	Adaptive Crested Porcupine Optimizer
CPR	Cyclic Population Reduction
LA-MPRS	Layer-Aware Microservice Placement and Request Scheduling
MDBPP	Multi-dimensional Bin Packing Problem
DQN	Deep Q-Network

## References

[1] Duan Q., Wang S., Ansari N. (2020). Convergence of networking and cloud/edge computing: Status, challenges, and opportunities. IEEE Netw..

[2] Tian X., Meng H., Shen Y., Zhang J., Chen Y., Li Y. (2024). Dynamic Microservice Deployment and Offloading for Things–Edge–Cloud Computing. IEEE Internet Things J..

[3] Shi Y., Yang Y., Yi C., Chen B., Cai J. (2024). Toward Online Reliability-Enhanced Microservice Deployment with Layer Sharing in Edge Computing. IEEE Internet Things J..

[4] Shi J., Fu K., Wang J., Chen Q., Zeng D., Guo M. (2024). Adaptive QoS-Aware Microservice Deployment with Excessive Loads via Intra- and Inter-Datacenter Scheduling. IEEE Trans. Parallel Distrib. Syst..

[5] Hu M., Wang H., Xu X., He J., Hu Y., Deng T., Peng K. (2024). Joint Optimization of Microservice Deployment and Routing in Edge via Multi-Objective Deep Reinforcement Learning. IEEE Trans. Netw. Serv. Manag..

[6] Zeng D., Geng H., Gu L., Li Z. Layered Structure Aware Dependent Microservice Placement Toward Cost Efficient Edge Clouds. Proceedings of the IEEE Conference on Computer Communications (IEEE INFOCOM 2023).

[7] Lv W., Wang Q., Yang P., Ding Y., Yi B., Wang Z., Lin C. (2022). Microservice deployment in edge computing based on deep Q learning. IEEE Trans. Parallel Distrib. Syst..

[8] Wang C., Yu H., Li X., Ma F., Wang X., Taleb T., Leung V.C.M. (2024). Dependency-Aware Microservice Deployment for Edge Computing: A Deep Reinforcement Learning Approach with Network Representation. IEEE Trans. Mob. Comput..

[9] Calcote L., Butcher Z. (2019). Istio: Up and Running: Using a Service Mesh to Connect, Secure, Control, and Observe.

[10] Hussain F., Li W., Noye B., Sharieh S., Ferworn A. Intelligent Service Mesh Framework for Api Security and Management. Proceedings of the 2019 IEEE 10th Annual Information Technology, Electronics and Mobile Communication Conference (IEMCON).

[11] Kang M., Shin J.S., Kim J. Protected Coordination of Service Mesh for Container-Based 3-Tier Service Traffic. Proceedings of the 2019 International Conference on Information Networking (ICOIN).

[12] Meng L., Sun Y., Zhang S. (2020). Capestor: A Service Mesh-Based Capacity Estimation Framework for Cloud Applications. Proceedings of the 13th International Conference on Cloud Computing (CLOUD 2020), Held as Part of the Services Conference Federation (SCF 2020).

[13] Ashok S., Godfrey P.B., Mittal R. Leveraging Service Meshes as a New Network Layer. Proceedings of the 20th ACM Workshop on Hot Topics in Networks.

[14] Chen L., Tang F., Li X., Liu J., Zhu Y., Yu J. (2024). Adaptive Network Management Service Based on Control Relation Graph for Software-Defined LEO Satellite Networks in 6G. IEEE Trans. Serv. Comput..

[15] Li Y., Zeng D., Chen L., Gu L., Ma W., Gao F. Cost Efficient Service Mesh Controller Placement for Edge Native Computing. Proceedings of the 2022 IEEE Global Communications Conference (GLOBECOM 2022).

[16] Kangjin W., Yong Y., Ying L., Hanmei L., Lin M. Fid: A Faster Image Distribution System for Docker Platform. Proceedings of the 2017 IEEE 2nd International Workshops on Foundations and Applications of Self* Systems (FAS* W).

[17] Huang Z., Wu S., Jiang S., Jin H. Fastbuild: Accelerating Docker Image Building for Efficient Development and Deployment of Container. Proceedings of the 2019 35th Symposium on Mass Storage Systems and Technologies (MSST).

[18] Zhao H., Deng S., Liu Z., Yin J., Dustdar S. (2019). Distributed redundant placement for microservice-based applications at the edge. arXiv.

[19] Peng K., He J., Guo J., Liu Y., He J., Liu W., Hu M. (2024). Delay-Aware Optimization of Fine-Grained Microservice Deployment and Routing in Edge via Reinforcement Learning. IEEE Trans. Netw. Sci. Eng..

[20] Li W., Li Z., Yan Z., Liu Y., Zeng D., Yu H., Chen W., Wu F. (2025). A data encryption and file sharing framework among microservices-based edge nodes with blockchain. Peer-to-Peer Netw. Appl..

[21] Sufiev H., Haddad Y., Barenboim L., Soler J. (2019). Dynamic SDN controller load balancing. Future Internet.

[22] Babayiğit B., Ulu B. (2021). A High Available Multi-Controller Structure for SDN and Placement of Multi-Controllers of SDN with Optimized K-means Algorithm. J. Inst. Sci. Technol..

[23] Naning H.S., Munadi R., Effendy M.Z. SDN Controller Placement Design: For Large Scale Production Network. Proceedings of the 2016 IEEE Asia Pacific Conference on Wireless and Mobile (APWiMob).

[24] Narantuya J., Yoon S., Lim H., Cho J.H., Kim D.S., Moore T., Nelson F. SDN-Based IP Shuffling Moving Target Defense with Multiple SDN Controllers. Proceedings of the 2019 49th Annual IEEE/IFIP International Conference on Dependable Systems and Networks–Supplemental Volume (DSN-S).

[25] Kelian V.H., Warip M.N.M., Ahmad R.B., Ehkan P., Zakaria F.F., Ilyas M.Z., Ng Y.P., Ikhwan A. (2024). An Efficacy of Multi-Controller Model Deployment Strategy in Software-Defined Networks. J. Adv. Res. Appl. Sci. Eng. Technol..

[26] Xiao J., Pan X., Liu J., Wang J., Zhang P., Abualigah L. (2024). Load balancing strategy for SDN multi-controller clusters based on load prediction. J. Supercomput..

[27] Noor A.K. (1981). Recent advances in reduction methods for nonlinear problems. Comput. Struct..

[28] Loiola E.M., de Abreu N.M.M., Boaventura-Netto P.O., Hahn P., Querido T. (2007). A survey for the quadratic assignment problem. Eur. J. Oper. Res..

[29] Su H., Zhao D., Heidari A.A., Liu L., Zhang X., Mafarja M., Chen H. (2023). RIME: A physics-based optimization. Neurocomputing.

[30] Abdel-Basset M., Mohamed R., Abouhawwash M. (2024). Crested Porcupine Optimizer: A new nature-inspired metaheuristic. Knowl.-Based Syst..

[31] Lai P., He Q., Abdelrazek M., Chen F., Hosking J., Grundy J., Yang Y. (2018). Optimal Edge User Allocation in Edge Computing with Variable Sized Vector Bin Packing. Proceedings of the Service-Oriented Computing: 16th International Conference (ICSOC 2018).

[32] Luo S., Xu H., Lu C., Ye K., Xu G., Zhang L., Ding Y., He J., Xu C. Characterizing Microservice Dependency and Performance: Alibaba Trace Analysis. Proceedings of the ACM Symposium on Cloud Computing.

[33] Wang S., Ding Z., Jiang C. (2020). Elastic scheduling for microservice applications in clouds. IEEE Trans. Parallel Distrib. Syst..

[34] Qu C., Calheiros R.N., Buyya R. (2018). Auto-scaling web applications in clouds: A taxonomy and survey. ACM Comput. Surv. (CSUR).

[35] Gu L., Zeng D., Hu J., Li B., Jin H. Layer Aware Microservice Placement and Request Scheduling at the Edge. Proceedings of the IEEE Conference on Computer Communications (IEEE INFOCOM 2021).

[36] Yu M., Yi Y., Rexford J., Chiang M. (2008). Rethinking virtual network embedding: Substrate support for path splitting and migration. ACM SIGCOMM Comput. Commun. Rev..

[37] Osband I., Blundell C., Pritzel A., Van Roy B. (2016). Deep exploration via bootstrapped DQN. arXiv.

